# Real World Comparison of Direct Selective Laser Trabeculoplasty Versus Selective Laser Trabeculoplasty: 12-Month Retrospective Study of a Tertiary Center in the UK

**DOI:** 10.3390/biomedicines14010156

**Published:** 2026-01-12

**Authors:** Piero Zollet, Federico Macario, Rachel Healy, Demetri T. Manasses, Rani T. Sebastian, Mario R. Romano

**Affiliations:** 1 University Hospital Wales, Cardiff CF14 4XW, UK; 2 Bristol Eye Hospital, Bristol BS1 2LX, UK; 3 Department of Biomedical Sciences, Humanitas University, Pieve Emanuele, 20090 Milan, Italy; 4 Department of Ophthalmology, Humanitas Gavazzeni-Castelli, 24128 Bergamo, Italy

**Keywords:** glaucoma, ocular hypertension, SLT

## Abstract

**Background:** Direct selective laser trabeculoplasty (DSLT) is a novel option for intraocular pressure (IOP) control in patients with glaucoma or ocular hypertension. The automated and touchless translimbal delivery of laser energy to 360 degrees of the trabecular meshwork (TM) improves aqueous outflow and lowers IOP. DSLT is faster, simpler, and less invasive than routinely performed SLT. Few studies have compared the two techniques. **Objective:** To retrospectively compare the safety and efficacy of DSLT and SLT over a 1-year follow-up period. **Methods:** In total, 16 eyes that underwent DSLT and 16 eyes that underwent SLT were included. The primary outcome measures were mean absolute and percent IOP reduction, number of medications, and BCVA at 1, 3, 6, and 12 months. Survival analysis on 1-year data was performed based on the presence of one or more of the following failure criteria: (1) IOP > 21 mmHg or less than 20% reduction in IOP from baseline at two consecutive visits; (2) increase in the number of IOP-lowering drops from baseline at two consecutive visits; (3) further procedures. **Results:** The survival rates in the DSLT vs. SLT group were 81% vs. 78%, 44% vs. 62%, and 37% vs. 43% at 3, 6, and 12 months, respectively. No statistically significant differences were reported. DSLT does not seem inferior to conventional SLT in terms of safety and efficacy in reducing IOP. **Conclusions:** The advantages of an automated, rapid, contactless technique may enlarge the cohort of patients eligible for a drop-free first-line IOP control procedure.

## 1. Introduction

Glaucoma is a chronic optic neuropathy characterized by damage to the optic nerve head (ONH) and progressive apoptosis of ganglionic retinal cells (GCL) and the retinal nerve fiber layer (RNFL). It leads to peripheral and occasionally central vision loss, and if untreated, it can cause irreversible blindness [1].

Elevated intraocular pressure (IOP), a crucial determinant of disease progression, remains the only modifiable risk factor; thus, all current treatments (medications, lasers, and surgery) aim to reduce the IOP [2].

The mainstay of the medical treatment of glaucoma has relied for decades on IOP-lowering eye drops as a first-line approach [3].

In recent years, multiple clinical studies reported efficacy in reducing IOP of laser trabeculoplasty as an initial or adjunctive treatment for primary open-angle glaucoma or ocular hypertension [4,5].

Selective laser trabeculoplasty (SLT) is a treatment that, instead of argon, uses a 532 nm frequency-doubled q-switched neodymium/yttrium–aluminum–garnet laser [6,7] and was endorsed by the United States Food and Drug Administration for the treatment of glaucoma in 2001 [4].

Laser trabeculoplasty lowers intraocular pressure by inducing biological changes in the trabecular meshwork (TM) resulting in increased aqueous outflow.

Treatment of early Primary Open Angle Glaucoma (POAG) with first-line SLT is an effective strategy lasting a period of several years. The potential for economic benefits in avoiding medications, and simultaneously improving quality of life in these cases is substantial [8].

In 2019, the Laser in Glaucoma and Ocular Hypertension (LiGHT) Trial demonstrated that SLT is at least as effective as eye drops and should be considered as a first-line treatment in patients with mild or moderate open-angle glaucoma or Ocular Hypertension (OHT) [9].

The American Academy of Ophthalmology Preferred Practice Pattern for POAG [10], the European Glaucoma Society (EGS) [11], and the National Institute for Health and Care Excellence (NICE) [12] guidelines indicate that SLT can be offered as an initial treatment or as an add-on treatment for POAG with a moderate level of evidence.

The EGS guidelines consider, with a weaker level of evidence, the indication of SLT even in patients with Pseudo-Exfoliative Glaucoma (PXFG) and Pigment Dispersion Glaucoma (PDG). At the same time, it is contraindicated in Neovascular, Uveitic, Post-Traumatic Glaucoma and with angle closure or angle dysgenesis.

Conventional SLT is performed using a gonioscopy lens placed on the patient’s cornea with a coupling agent, which enables the beam to be aimed directly at the TM. SLT is performed with a variable number of laser spots on 180 or 360 degrees of the TM, and the laser power is titrated according to the treatment protocol used: standard, just below the cavitation bubble level, or high, producing bubbles at 50% or more of the laser spots [13].

Visualization of the angle with a gonioscopy lens requires sufficient angle width. The procedure requires training and experience, which challenges many ophthalmologists [14]. It is not exempt from complications: a transient elevation of IOP, mild inflammation (iritis), and corneal endothelial damage may occur [15].

A newer and simpler technique has been recently developed to stimulate the TM in a less invasive manner. Direct selective laser trabeculoplasty (DSLT) is an automated procedure performed directly through the limbus without gonioscopy. The principle underlying DSLT relies upon a laser beam with a translimbal energy delivery to the TM to achieve an improvement in the aqueous outflow and an IOP-lowering effect without any contact with the patient’s eye [16]. An eye speculum is placed upon the patient’s eyelid. Then, an eye-tracking system automatically identifies the target area on the limbus and maintains the target when the eye is moving. Finally, a predefined number of laser pulses with a certain level of energy is delivered through a full 360 degrees around the limbus to the TM.

Compared to conventional SLT, DSLT is more rapid, taking around 2 s. It does not depend on the operator’s experience, and it does not require any contact. As a result, the risk of corneal infection or damage is virtually absent [17].

In 2017, Geffen et al. [16] were the first to publish the results of a trial reporting equal efficacy at 1 year between conventional SLT and DSLT in terms of safety and IOP reduction. In 2021, the first clinical trial of automated DSLT for patients with OAG demonstrated that translimbal DSLT in patients treated with a preset energy level of ≥1 mJ did not differ significantly from the reported IOP reduction obtained 6 months post SLT treatment in prospective randomized clinical trials [17,18].

This study aims to assess the hypothesis that treatment by a new automated device for DSLT is no worse than that of conventional SLT and to determine whether it is effective in reducing IOP.

## 2. Methods

### 2.1. Study Design

This was a retrospective single-center case series of patients diagnosed with glaucoma or ocular hypertension who underwent either conventional SLT or automated DSLT between August 2020 and August 2023 at Bristol Eye Hospital, Bristol, UK. An exemption, given the retrospective design, from full institutional review board approval was obtained from the ethical committee of the University Hospitals Bristol and Weston NHS Foundation Trust.

### 2.2. Patients

The patients included in this study were treated with either SLT or automated direct SLT for glaucoma or OHT. The inclusion criteria were patients over the age of 18 years who had poorly controlled glaucoma and/or demonstrated noncompliance or intolerability to topical hypotensive treatment, and treatment-naive patients. Exclusion criteria were history of previous glaucoma surgeries or previous SLT/ALT treatment, and best corrected visual acuity (BCVA) lower than 20/200. Patients with contraindications to SLT were excluded, as were patients with angle closure glaucoma, congenital dysgenesis, or secondary glaucoma (except exfoliative or pigmentary glaucoma).

### 2.3. Procedure

All the patients included in this study had an up-to-date full ophthalmic examination including visual acuity, IOP check, slit lamp exam, gonioscopy, and visual fields conducted before the treatment.

The patients were pre-medicated with pilocarpine 2% and aproclonidine 0.5% 15–30 min before the laser treatment. The patients in the SLT group were treated with 360° SLT using 100 spots distributed equally in the four quadrants and using a laser energy titrated starting from 0.8 mJ to achieve a ‘champagne bubbles’ reaction in approximately 50% of shots. The lens used was the Latina lens (Volk Ltd., Mentor, OH, USA), while the laser was a Selector II combined SLT/YAG (Litechnica Ltd., London, UK).

Automated direct SLT patients were treated with 120 spots and delivered over 360 degrees of the perilimbal sclera with a power set according to consultant clinical judgment between 1.0 and 1.8 mJ per spot after a dose response trial was performed. The machine used was the Belkin Eagle (Belkin Lasers Ltd., El Segundo, CA, USA). The automated DSLT device employs a Q-switch, frequency-doubled Nd:YAG laser with a wavelength of 532 nm. It directs a 7 ns pulse, 400 μm laser beam directly to the perilimbal sclera without needing a gonioscopic lens.

Eye tracking compensates for eye movements and delivers the treatment spots in less than 2.5 s. The DSLT and the SLT procedures were carried out either by or under the supervision of two senior glaucoma consultants (D.T.M., R.T.S.).

A slit lamp examination of the anterior segment was conducted in both groups just after the procedure to look for any sign of complications, and the IOP check was repeated 30 min after the procedure to detect any possible IOP spike.

Patients received a follow-up appointment including complete ophthalmic examination including visual acuity, IOP check with Goldman applanation tonometry, slit lamp exam at Bristol Eye Hospital at 1 month, 3 months, 6 months, and 12 months after the procedure.

### 2.4. Outcome Measures

The primary outcome measures were mean absolute and percent IOP reduction, number of medications, and BCVA at 1, 3, 6, and 12 months. Secondary outcomes were survival analysis results, complication, and retreatment type and rate. Survival analysis on 1-year data was performed based on the presence of one or more of the following failure criteria: (1) IOP > 21 mm Hg or less than 20% reduction in IOP from baseline at two consecutive visits; (2) increase in the number of IOP-lowering drops from baseline at two consecutive visits; (3) further glaucoma or laser procedures. Intuitively, success was defined as a reduction in IOP ≥ 20% without any additional medication or any additional glaucoma procedure.

### 2.5. Statistical Methods

The data were gathered from the hospital electronic medical record system (Medisoft Ltd., Leeds, UK) and entered in a pseudonymized Excel spreadsheet (Microsoft Corp. Redmond, Washington, DC, USA), and the statistical analysis was conducted using IBM SPSS software for Windows (version 28.0, IBM Corp., Armonk, NY, USA). A *p*-value of less than 0.05 was considered as the threshold of significance.

The Shapiro–Wilk test was used to check if continuous numerical variables followed a normal distribution. Since not all the samples were normally distributed, to perform the comparisons and determine if any statistically significant difference was present between the samples we employed the Wilcoxon rank sum test with continuity correction.

If the patient had further laser or other surgery for glaucoma in the same eye, data were censored at the date of the additional procedure. Patients who died or who were lost to follow-up were omitted from the IOP, BCVA, and medication analysis at these timepoints and censored for the purpose of the survival analysis. Kaplan–Meier plots were used to demonstrate survival and the 95% confidence intervals were computed.

## 3. Results

### 3.1. Demographics

In the direct SLT group a total of 16 eyes and 9 patients were included. In the SLT group a total of 16 eyes and 9 patients were included.

Table 1 presents a summary of demographics and baseline characteristics.

### 3.2. Outcomes and Comparisons

Data regarding the mean IOP, number of medications, visual acuity, and need for further procedures are summarized in Table 2. The *p*-values represent the significance of the difference between the SLT and DSLT distributions at any specific time point.

In the DSLT group, the baseline mean IOP was 21.77 (±5.39) mmHg. The mean IOP, excluding failures, was 17.45 (±6.13) at 1 month, 18.23 (±5.21) at 3 months, 17.73 (±5.35) at 6 months, and 15.57 (±4.20) at 12 months. The number of antiglaucoma drugs decreased from an average of 1.19 (±0.39) preoperatively to 1.08 (±0.49) at 1 month, 1.62 (±0.96) at 3 months, 1.47 (±0.92) at 6 months, and 1.0 (±0.78) at 12 months.

In the SLT group, the baseline mean IOP was 21.69 (±4.05) mmHg. The mean IOP, excluding failures, was 18.40 (±5.68) at 1 month, 16.9 (43.65) at 3 months, 17.22 (±3.23) at 6 months, and 18.36 (±3.89) at 12 months. The number of antiglaucoma drugs decreased from an average of 1.25 (±0.75) preoperatively to 1.1 (±0.74) at 1 month, 0.73 (±0.64) at 3 months, 1.00 (±0.73) at 6 months, and 1.0 (±0.78) at 12 months. No statistical differences between the two groups were detected except for the number of glaucoma medications at 3 months (*p* < 0.01), with a significant higher mean number in the DSLT patients.

In the DSLT group, the treatment success was 81% at 3 months, declining to 44% and 38% at 6 and 12 months post DSLT, respectively. For all eyes that failed DSLT before the last follow-up assessment (*n* = 10), the reasons for failure that were evident on the date of failure were as follows: further glaucoma procedure required (*n* = 3), IOP > 21 mmHg (*n* = 3), IOP reduction < 20% (*n* = 1), and increase in number of medications (*n* = 4).

In the SLT group, the treatment success was 78% at 3 months, declining to 62% and 43% at 6 and 12 months post SLT, respectively. For all eyes that failed SLT before the last follow-up assessment (*n* = 9), the reasons for failure that were evident on the date of failure were as follows: further glaucoma procedure required (*n* = 0), IOP > 21 mmHg (*n* = 3), IOP reduction < 20% (*n* = 8), and increase in number of medications (*n* = 0).

Given that patients may have satisfied more than one failure criterion on the failure date, the percentages add up to a greater number than each group’s actual failures.

No complications were observed in the SLT and DSLT groups.

According to the Shapiro–Wilk test, IOP, VA, and number of medications samples at the different time points were not constantly normally distributed.

The Wilcoxon test demonstrated that the change in the number of medications was statistically different only at 3 months between the DSLT and SLT groups.

**Figure 1:**
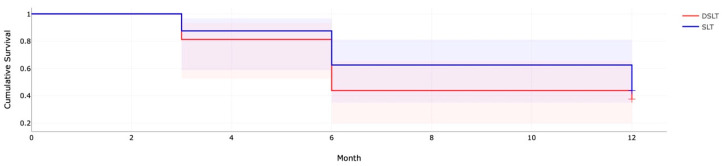
Kaplan–Meier survival analysis comparing cumulative treatment success following selective laser trabeculoplasty (SLT) and direct selective laser trabeculoplasty (DSLT) over a 12-month follow-up period. SLT demonstrates higher cumulative success rates compared with DSLT throughout follow-up. Shaded regions indicate 95% confidence intervals, and stepwise declines reflect treatment failures over time.

The Kaplan–Meier survival analysis results are summarized in Figure 1 and Table 3. The two curves were not statistically different from each other (*p* = 0.57); however, a larger proportion of failures at 6 and 12 months were detected in the DSLT group.

## 4. Discussion

The safety and efficacy of SLT have been widely demonstrated in different studies for various types of glaucoma as well as in ocular hypertension [18]. Moreover, the advantage in terms of cost-effectiveness and quality of life of SLT treatment versus chronic use of IOP-lowering drugs has been documented by various sources [8,9]. In particular, the LiGHT (Laser in Glaucoma and ocular HyperTension) study validated its application over medical therapies as a primary treatment capable of managing IOP at a lower cost and with fewer side effects. Nevertheless, the widespread adoption of SLT treatment in real-life settings is still limited due to the skills and equipment required to perform it and to the reluctance of some ophthalmologists to prescribe laser procedures as a first step in treating glaucoma or ocular hypertension [8].

Our study represents one of the few studies to date comparing the efficacy of DSLT with SLT in managing glaucoma and OHT. In our setting, most eyes initially responded to SLT and DSLT treatment. We have reported a similar percentage of success defined as a reduction in IOP ≥ 20% without any additional medication or any additional glaucoma procedure both in the DSLT group (81%, 44%, and 38% at 3, 6, and 12 months, respectively) and SLT group (78%, 62%, and 43% at 3, 6, and 12 months, respectively). However, in both groups, a large number had failed treatment by 1 year because of an inadequate reduction in IOP (>21 mmHg or <20% reduction) or an increase in the number of glaucoma medications or by undergoing a subsequent glaucoma procedure. In a large retrospective analysis involving patients who had undergone SLT in the UK, the treatment success, defined with the same criteria applied in our study, was 70% at 6 months and 45% at 12 months, consistent with our findings.

The first prospective trial [17] to validate DSLT efficacy reported an overall mean IOP reduction in 15 eyes of 18.1%, 21.4%, and 18.8% at 1, 3, and 6 months, respectively. In our study, an overall mean IOP reduction of 21.6%, 24.4%, and 11% was reported at 1, 3, and 6 months in the SLT group and 13.2%, 9.4%, and 14.8% in the DSLT group. Excluding failures, the mean percentage reduction in IOP in the DSLT group was 23.50% (±4.94) versus 20.90% (±11.0) in the SLT group at 12 months. Geffen et al. reported an average IOP reduction after 6 and 12 months of 23.4% and 20.8% for the DSLT group and 27.1% and 33.7% for the SLT group [16].

A retrospective analysis recently published by Fossati et al. [19] reported a percentage of success of 73% with a mean reduction in IOP of 25.7% in a group of 15 eyes treated with DSLT and followed up for 4 months. 

The recent results of the GLAUrious Study, a prospective, multicenter, randomized, controlled, evaluator-masked noninferiority trial that represents one of the most significant studies on DSLT, have been published [20]. The GLAUrious study failed to demonstrate that DSLT was noninferior to SLT. At 6 months, the mean washout IOP reduction from baseline was 5.5 ± 0.5 mmHg (−20.6%) following DSLT and 6.2 ± 0.5 mmHg (−23.6%) following SLT (*p* = 0.09). In our cohort, at 6 months, the mean IOP reduction was 4.0 ± 0.7 mmHg (−18.5%) in the DSLT group and 4.4 ± 0.6 mmHg (−20.3%) in the SLT group (*p* = 0.79). Consistent with the GLAUrious Study, the IOP reduction was slightly greater in patients undergoing SLT, both in absolute and relative terms. It should be emphasized that, in our protocol, patients who were already receiving topical glaucoma medications did not undergo a washout period before treatment initiation. However, DSLT did provide effective IOP reduction for 12 months in both studies [20].

Our findings are similar to previous results in the existing literature in terms of safety and efficacy. The advantage of this novel approach includes the possibility of treating narrow iridocorneal angles that are difficult to visualize, and of patients with unfavorable anatomic characteristics or patients who are not compliant with the gonioscopy lens applied to their eyes during a conventional SLT [21]. The absence of contact between the lens and the cornea reduces the risk of cornea-related complications [19].

In conclusion, DSLT may provide a valid alternative to SLT in managing POAG and OHT. This study supports the hypothesis that treatment by an automated device for DSLT is not inferior to that of conventional SLT in terms of safety and efficacy in reducing intraocular pressure.

Our study has several limitations, including the limited sample size and the relative diversity of the cohort of patients included in this study, which varied, among other factors, for use or not of antiglaucoma medications before treatment. One strength of this study is the length of the follow-up period, which is longer compared to similar studies available in the literature. Large randomized, controlled clinical trials are needed to expand the knowledge on the role that DSLT could play in the treatment setting of POAG and OHT.

## Figures and Tables

**Table 1:** Baseline demographic and clinical characteristics for 32 patient eyes undergoing selective laser trabeculoplasty or direct selective laser trabeculoplasty. (Abbreviations: DSLT direct selective laser trabeculoplasty; IOP intraocular pressure; MD mean deviation; OAG open-angle glaucoma; OHT ocular hypertension; PSD pattern standard deviation; PXG pseudo-exfoliative glaucoma; SLT selective laser trabeculoplasty; VA visual acuity; VFI visual field index).

Parameter	DSLT	SLT	*p*-Value
Patients n°	9	9	1
Eyes n°	16	16	1
Right (%)	50%	44%	1
Males (%)	50%	50%	1
Age (Mean ± SD)	73.62 (5.35)	75.06 (6.68)	0.78
Ethnicity			
Caucasian n° (%)	16 (100%)	16 (100%)	1
Diagnosis n° (%)	16 (100%)	16 (100%)	
POAG	10 (63%)	3 (19%)	0.02
OHT	6 (37%)	11 (69%)	0.02
PXG	0 (0%)	2 (12%)	0.02
Baseline drops n° (%)			
0	0	3	0.19
1	13	6	0.19
2	3	7	0.19
Baseline drops (Mean ± SD)	1.19 (0.39)	1.25 (0.75)	0.38
Diamox	0	0	1
IOP baseline (Mean ± SD)	21.77 (5.39)	21.69 (4.05)	0.98
VA baseline LogMar (Mean ± SD)	0.10 (0.13)	0.09 (0.17)	0.95
Visual field (Mean ± SD)			
VFI	94.12 (6.10)	96.31 (3.11)	0.50
MD	−0.50 (3.84)	−1.07 (2.01)	0.78
PSD	3.81 (3.84)	2.35 (1.10)	0.46
Adverse events			
N°			
IOP spike	0	0	1
Other	0	0	1

**Table 2:** Outcome and comparison data regarding intraocular pressure (IOP), number of drops, visual acuity (VA), and additional glaucoma procedures between DSLT and SLT eyes. The mean value with its respective standard deviation (SD) in brackets is reported in each box. *p*-value is highlighted when considered statistically significant (*p* < 0.05).

				DSLT				SLT		
Time	Baseline	1 mo	3 mo	6 mo	12 mo	Baseline	1 mo	3 mo	6 mo	12 mo
IOP (mmHg) (Mean ± SD)	21.77 (5.39)	17.45 (6.13)	18.23 (5.21)	17.73 (5.35)	15.57 (4.20)	21.69 (4.05)	18.4 (5.68)	16.9 (3.65)	17.22 (3.23)	18.36 (3.89)
*p*-value		0.71	0.26	0.79	0.42		0.71	0.26	0.79	0.42
N° drops (Mean ± SD)	1.19 (0.39)	1.08 (0.49)	1.62 (0.96)	1.47 (0.92)	1.00 (0.78)	1.25 (0.75)	1.1 (0.74)	0.73 (0.64)	1.0 (0.73)	1.0 (0.78)
*p*-value		0.69	0.01	0.20	0.54		0.69	0.01	0.20	0.54
VA (LogMar) (Mean ± SD)	0.10 (0.13)	0.07 (0.06)	0.07 (0.07)	0.08 (0.10)	0.12 (0.14)	0.09 (0.17)	0.07 (0.009)	0.05 (0.07)	0.11 (0.16)	0.08 (0.12)
*p*-value		0.65	0.92	0.92	0.41		0.65	0.92	0.92	0.41
Additional glaucoma procedures (n°)		0	1	2	0		0	0	0	0
Repeated SLT	-	0	0	2	0	-	0	0	0	0
Preserflo	-	0	1	0	0	-	0	0	0	0

n° = number.

**Table 3:** Survival analysis of treatment success with respective failure criteria.

		Baseline	1 Month	3 Months	6 Months	12 Months	Total
SLT	SURVIVOR	16	16 (100%)	14 (88%)	10 (63%)	7 (44%)	7
	FAILURE		0	2	4	3	9
	Need for a glaucoma procedure		/	/	/	/	
	IOP > 21 mmHg from baseline on any 2 consecutive visits or IOP reduction < 20% from baseline on any 2 consecutive visits		/	2	4	3	
	Increase in the number of antiglaucoma medications baseline on any 2 consecutive visits		/	/	/	/	
DSLT	SURVIVOR	16	16 (100%)	13 (81%)	7 (44%)	6 (38%)	6
	FAILURE		/	3	6	1	10
	Need for a glaucoma procedure		/	1	2	/	
	IOP > 21 mmHg from baseline on any 2 consecutive visits or IOP reduction < 20% from baseline on any 2 consecutive visits		/	2	2	1	
	Increase in the number of antiglaucoma medications baseline on any 2 consecutive visits		/	/	2	/	

## Data Availability

The original contributions presented in this study are included in the article material. Further inquiries can be directed to the corresponding author.

## References

[1] Stein J.D., Khawaja A.P., Weizer J.S. (2021). Glaucoma in Adults-Screening, Diagnosis, and Management: A Review. JAMA.

[2] Wagner I.V., Stewart M.W., Dorairaj S.K. (2022). Updates on the Diagnosis and Management of Glaucoma. Mayo. Clin. Proc. Innov. Qual. Outcomes.

[3] Weinreb R.N., Aung T., Medeiros F.A. (2014). The pathophysiology and treatment of glaucoma: A review. JAMA.

[4] Gračner T. (2019). Comparative study of the efficacy of selective laser trabeculoplasty as initial or adjunctive treatment for primary open-angle glaucoma. Eur. J. Ophthalmol..

[5] Gazzard G., Konstantakopoulou E., Garway-Heath D., Barton K., Wormald R., Morris S., Hunter R., Rubin G., Buszewicz M., Ambler G. (2023). Laser in Glaucoma and Ocular Hypertension (LiGHT) Trial. Ophthalmology.

[6] Latina M.A., Sibayan S.A., Shin D.H., Noecker R.J., Marcellino G. (1998). Q-switched 532-nm Nd:YAG laser trabeculoplasty (selective laser trabeculoplasty): A multicenter, pilot, clinical study. Ophthalmology.

[7] Juzych M.S., Chopra V., Banitt M.R., Hughes B.A., Kim C., Goulas M.T., Shin D.H. (2004). Comparison of long-term outcomes of selective laser trabeculoplasty versus argon laser trabeculoplasty in open-angle glaucoma. Ophthalmology.

[8] Ansari E. (2021). 10-year outcomes of first-line selective laser trabeculoplasty (SLT) for primary open-angle glaucoma (POAG). Graefes. Arch. Clin. Exp. Ophthalmol..

[9] Gazzard G., Konstantakopoulou E., Garway-Heath D., Garg A., Vickerstaff V., Hunter R., Ambler G., Bunce C., Wormald R., Nathwani N. (2019). Selective laser trabeculoplasty versus eye drops for first-line treatment of ocular hypertension and glaucoma (LiGHT): A multicentre randomised controlled trial. Lancet.

[10] AAO PPP Glaucoma Committee, Hoskins Center for Quality Eye Care Primary Open-Angle Glaucoma PPP 2020. https://www.aao.org/education/preferred-practice-pattern/primary-open-angle-glaucoma-ppp.

[11] European Glaucoma Society (2020). Terminology and Guidelines for Glaucoma 5th. https://eugs.org/educational_materials/6.

[12] (2017). Overview|Glaucoma: Diagnosis management|NICE Guidance. https://www.nice.org.uk/guidance/ng81.

[13] Dahlgren T., Ayala M., Zetterberg M. (2024). Optimal Performance of Selective Laser Trabeculoplasty: Results from the Swedish Optimal SLT Multicenter Randomized Controlled Trial. Ophthalmol. Glaucoma.

[14] Lieberman M.F., Congdon N.G., He M. (2011). The value of tests in the diagnosis and management of glaucoma. Am. J. Ophthalmol..

[15] Odberg T., Sandvik L. (1999). The medium and long-term efficacy of primary argon laser trabeculoplasty in avoiding topical medication in open angle glaucoma. Acta Ophthalmol. Scand..

[16] Geffen N., Ofir S., Belkin A., Segev F., Barkana Y., Kaplan Messas A., Assia E.I., Belkin M. (2017). Transscleral Selective Laser Trabeculoplasty Without a Gonioscopy Lens. J. Glaucoma.

[17] Goldenfeld M., Belkin M., Dobkin-Bekman M., Sacks Z., Meirovitch S.B., Geffen N., Leshno A., Skaat A. (2021). Automated Direct Selective Laser Trabeculoplasty: First Prospective Clinical Trial. Transl. Vis. Sci. Technol..

[18] Katz L.J., Steinmann W.C., Kabir A., Molineaux J., Wizov S.S., Marcellino G. (2012). Selective laser trabeculoplasty versus medical therapy as initial treatment of glaucoma: A prospective, randomized trial. J. Glaucoma.

[19] Fossati G., Trevisi M., Sarodia U., Malick H., Abdou H., Sodeinde M., Romano M.R., Osman L. (2025). Direct selective laser trabeculoplasty: A retrospective study. Eur. J. Ophthalmol..

[20] Gazzard G., Congdon N., Azuara-Blanco A., Blumenthal E.Z., Gomelauri K., Zaliniyan M., Traverso C.E., Bracha Z., Dvalishvili A., Solberg Y. (2025). Randomized Noninferiority Trial of Direct Selective Laser Trabeculoplasty in Open-Angle Glaucoma and Ocular Hypertension: GLAUrious Study. Ophthalmology.

[21] Sacks Z.S., Dobkin-Bekman M., Geffen N., Goldenfeld M., Belkin M. (2020). Non-contact direct selective laser trabeculoplasty: Light propagation analysis. Biomed. Opt. Express..

